# Insights into how manual therapists incorporate the biopsychosocial-enactive model in the care of individuals with CLBP: a qualitative study

**DOI:** 10.1186/s12998-025-00574-3

**Published:** 2025-02-18

**Authors:** Marco Bianchi, Giacomo Rossettini, Francesco Cerritelli, Jorge E. Esteves

**Affiliations:** 1 Malta ICOM Educational, Gzira, Malta; 2 Foundation COME Collaboration, Clinical-Based Human Research Department, Pescara, Italy; 3 School of Physiotherapy, University of Verona, Via Bengasi 4, Verona, 37134 Italy; 4 Department of Physiotherapy, Faculty of Sport Sciences, Universidad Europea de Madrid, Villaviciosa de Odón, Spain; 5 NYIT College of Osteopathic Medicine, Old Westbury, NY 11568 USA; 6 Escola Superior de Saúde Atlântica, Barcarena, Portugal

**Keywords:** Biopsychosocial model, Biopsychosocial-enactive model, Chronic low back pain, Manual therapy, Grounded theory, Person-centred care

## Abstract

**Background:**

Chronic low back pain (CLBP) presents a significant challenge for manual therapists. Recent advancements in pain research have highlighted the limitations of the traditional biomedical and biopsychosocial models, prompting the exploration of alternatives. The biopsychosocial-enactive (BPS-E) model has emerged as a promising alternative. This study aims to explore the application of the BPS-E model by manual therapists in managing CLBP and to initiate a meaningful dialogue about its use.

**Methods:**

This study adhered to the Standards for Reporting Qualitative Research. Guided by constructivist grounded theory, we conducted semi-structured interviews with ten manual therapists who are experts in the BPS-E model. Data collection, conceptualization, and analysis were systematically carried out to identify key themes and insights.

**Results:**

The core theme identified was “The person-centred approach,” with three subthemes: “Opportunities in implementing the model”, “Utilizing and Integrating Diverse Skills for Holistic Care”, and “Challenges in implementing the model”.

**Conclusion:**

This study provides insights into how manual therapists incorporate the BPS-E model in their practice, demonstrating its advantages over the traditional biopsychosocial model. The findings highlight the need for further research and training to effectively implement the BPS-E model in clinical settings. This research begins an essential discussion on the potential of the BPS-E model to enhance care for CLBP patients.

## Introduction

Chronic low back pain (CLBP) is a prevalent condition that affects approximately 30% of adults globally and is a leading cause of disability, creating significant burdens for healthcare systems worldwide [1–3]. Due to the increased demand for general practitioners (GPs) and longer wait times, manual therapists have become a crucial point of contact for individuals seeking treatment for CLBP [4, 5]. While traditional biomedical treatments, such as manual therapy and exercise, have been effective in alleviating symptoms, there is growing recognition that these approaches alone are insufficient for managing the complex nature of chronic pain [6–9].

The biopsychosocial (BPS) model, introduced by George Engel, was a major advancement in acknowledging the multifactorial nature of pain, incorporating biological, psychological, and social factors into its understanding [10]. However, despite its potential, the BPS model has faced criticisms regarding its practical application [11, 12]. In many clinical settings, it is applied in a fragmented and reductionist manner, where biological factors are often prioritised over psychological and social ones [13, 14]. This distorted application reduces the model’s capacity to offer truly holistic care [15–18]. Moreover, Engel’s model lacks a clear framework for integrating these dimensions, often resulting in a mechanistic and compartmentalised approach to pain management [19].

Recent critiques of the BPS model highlight its reductionist tendencies, with healthcare professionals frequently dividing pain into discrete biological, psychological, or social categories, rather than recognising it as a dynamic and interconnected phenomenon [12]. This compartmentalisation fails to address the complexities of pain as a lived experience, reducing human suffering to linear and isolated processes [18, 19]. Furthermore, there are persistent misconceptions regarding the BPS model, with some therapists viewing it as overly time-consuming or as primarily addressing mental health, which further limits its effectiveness [11, 13, 15, 18–22].

In response to these limitations, scholars have advocated for the integration of the enactive perspective into the BPS framework, offering a more cohesive and dynamic model of pain [12, 18, 19, 23, 24]. Enactivism, rooted in embodied cognition, suggests that pain is not simply located within the body but arises from the interaction between individuals and their environments [18]. This perspective shifts the focus from static biological markers to a relational understanding of pain as an emergent, embodied process. By incorporating the enactive perspective, the BPS model can evolve into a biopsychosocial-enactive (BPS-E) model, which emphasises the continuous interaction between body, mind, and environment [12, 18, 19, 23–25]. This framework captures the complexity of chronic pain more effectively, avoiding the pitfalls of reductionism and compartmentalisation that plague the traditional BPS model.

The BPS-E model offers several advantages over the conventional BPS framework. First, it provides a more integrative view by treating biological, psychological, and social factors as interconnected and inseparable components of the pain experience [12, 18, 25]. Second, it emphasises the role of lived experience and sense-making in shaping pain, moving beyond the simplistic notion that pain is purely a biological process [13]. This perspective aligns with contemporary theories of pain that highlight its embodied, extended, and emergent nature. Lastly, the BPS-E model addresses the existential dimension of pain, recognising the importance of individual values, goals, and sense of meaning in shaping both the experience of pain and treatment outcomes [19].

Despite its promise, the BPS-E model has yet to be fully integrated into clinical practice, particularly in manual therapy settings. This study seeks to address this gap by exploring how manual therapists incorporate the enactive dimension of the BPS model in their treatment of individuals with CLBP. By investigating the insights and experiences of these practitioners, the research aims to enhance our understanding of how the BPS-E model can be practically applied, ultimately leading to more holistic and effective approaches to pain management.

## Methods

### Study design

This qualitative study was designed by expert researchers (GR; FC; JE) with extensive clinical and methodological experience in manual therapy and a track record of publications in peer-reviewed journals [26, 27], and substantial expertise in qualitative research methodologies, including designing and conducting interviews, thematic analysis, and interpretation of qualitative data. The study received approval from the Malta ICOM Educational Ethics Committee (20/03/2022 N. BM000800CBFT) and complies with the Declaration of Helsinki [28]. The reporting of this study adhered to the Consolidated Criteria for Reporting Qualitative Research (COREQ) Checklist [29] to ensure transparency and comprehensiveness in the study’s methodology and findings.

The use of constructivist grounded theory was selected as the study’s methodological framework due to its ability to explore and construct meaning from complex social processes and interactions that underpin clinical practice [30]. This approach is particularly well-suited for examining the dynamic, context-dependent nature of clinical decision-making, as it allows for the co-construction of knowledge between the researchers and participants. By focusing on the lived experiences and perspectives of manual therapists, this methodology facilitates a deeper understanding of how the enactive-biopsychosocial model is applied in practice. Additionally, constructivist grounded theory supports the generation of evidence-based insights that align with the iterative and interpretive nature of clinical reasoning, making it a robust choice for generating actionable findings in healthcare research [31].

This methodology ensured that the study could capture the complex, evolving nature of manual therapists’ interactions with patients, making it ideally suited to inform evidence-based practice in managing chronic low back pain.

## Participants

### Recruitment

The authors employed a snowball sampling method [32] to identify manual therapists with experience in integrating the enactive dimension of the BPS model for managing CLBP. Initially, two authors (MB and JE) contacted a small group of known practitioners who had demonstrated relevant experience in integrating the enactive dimension in the BPS model. These practitioners were then asked to recommend other therapists who met the criteria for expertise in this model, but who varied in professional roles (e.g., physiotherapists, osteopaths, chiropractors, massage therapists), and practice settings (e.g., private clinics, hospitals, academic institutions). This referral process gradually expanded the pool of participants, ensuring the inclusion of highly qualified professionals with deep experience in applying the BPS-E model to CLBP.

In line with the concept of “information power,” which suggests that a smaller, well-targeted sample can provide sufficient insights when participants’ knowledge is highly relevant [33], a sample size of 8 to 12 participants was deemed appropriate. This range was considered adequate to capture a diverse array of perspectives while facilitating an in-depth exploration of the research topic.

Manual therapy was defined as a clinical approach involving hands-on techniques for the diagnosis and treatment of musculoskeletal conditions [34]. Participants were selected based on their formal training in manual therapy, clinical experience (minimum five years), ongoing practice, and demonstrated expertise in the BPS-E model through teaching, research, or clinical application. Included professions were physiotherapists, osteopaths, chiropractors, and massage therapists. Participants were chosen for their knowledge, ability to convey competencies, and significant clinical expertise [35, 36]. While these criteria ensured a high level of knowledge and experience, specific details on how participants were trained in the application of the BPS-E model are not available. This may be attributed to the recent application of the enactivist framework to the BPS model in general and MSK care, in particular. Exclusion criteria included therapists who did not meet the inclusion criteria, those with a personal relationship with the interviewer, and professionals whose practice was not directly centred on manual therapy. The researcher (MB) emailed 20 manual therapists, providing an overview of the study objectives, methods, and role requirements, along with a request for participation. Interested participants were sent a separate file containing written informed consent for the use of personal data and were required to confirm correspondence with the inclusion criteria. All who expressed interest ultimately participated in the study.

### Interviews

Interviews were conducted in either Italian or English, based on participants’ preferences, by MB, a male Italian osteopath with experience in qualitative research. To ensure impartiality, the principal researcher did not have any personal relationship with the interviewees.

### Design of the interview guide

A semi-structured interview approach was employed to gather data [37], allowing for flexibility in exploring emerging themes while maintaining a structured data collection approach [38]. Initial interview questions were developed, evaluated, and refined by researchers GR, FC, and JE [26, 27]. The guide was further refined through critical discussions with the research team and piloted with educators not involved in the study. Feedback from the pilot interviews led to iterative revisions of the interview guide, enabling the primary researcher to explore emerging themes from previous interviews and test their insights and hypotheses. The first question aimed to establish a comfortable dialogue, encouraging participants to express their thoughts and experiences candidly [38]. Subsequent questions delved deeper into the practitioners’ experiences and understanding of the topic, with follow-up prompts provided to elicit detailed responses [39] (Table 1).

**Table 1:** Semi-structured interview guide

Interview questions and prompts (Revised following analysis)
**What is your approach to the management of patients with chronic low back pain?** •What role do manual therapy, education, and exercises have in your daily practice?
Could you talk about your understanding of the BPS-E model? •What are its strengthst? •In your personal opinion and expertise, what are the limitations of the BPS-E model and the challenges associated with its implementation in patient care?
**Could you talk about your understanding of the BPS-E model?** • What are its strengthst? • In your personal opinion and expertise, what are the limitations of the BPS-E model and the challenges associated with its implementation in patient care? • Is there resistance to change from the biomedical approach? • What is the role of “biology”?
**How does the BPS-E model inform the treatment and management of chronic pain**,** including**,** for example**,** the role in modulating pain**,** altering neuroplastic changes**,** and changing existing priors and beliefs?** **•**Which strategy do you use to reassure patients of their condition and to communicate with them?
**Which are the social and psychological aspects that you consider in your case history? How do you assess these variables?** • What do psychosocial risk factors mean to you? • How do you measure the evolution of these parameters?
**How to use the BPS-E model in manual therapy?** **•**What are the perceptions of patients regarding this topic?
**Do you consider the use of the BPS-E model as an added value in your clinical practice? If yes**,** why?** **•**How have the result of your treatments changed since the use of the BPS-E model? •May you give some clinical examples?
**Would you like to add some comments that were not explored in the topics of previous questions?**
**The transcript will be sent to you to check. Thank you for participating**

Legend: Biopsychosocial-enactive (BPS-E); Chronic low back pain (CLBP)

Interviews were conducted online via Zoom between May and June 2022, recorded, and transcribed verbatim by the principal researcher (MB) to ensure accuracy [40]. The use of Zoom provided privacy, flexibility, and a familiar environment for participants, which is favored in qualitative research [41, 42]. Interviews were conducted individually to avoid conditioning [42]. Data collection adhered to the principles of constructivist grounded theory, emphasizing an iterative and collaborative approach to data gathering and analysis [43]. Data collection continued until saturation was reached, defined as when new themes generated fell below 5% [42]. Participants were allowed to edit, add, or remove any information to further enhance data credibility [44].

### Data analysis

To align with the principles of constructivist grounded theory while maintaining rigour, the authors employed a reflective approach throughout the data collection and analysis process. This process began during the interviews, which were designed as dynamic, interactive dialogues rather than rigidly structured sessions. Participants were encouraged to elaborate on their experiences and, in doing so, actively shape the direction of the discussion. This approach acknowledged the participants as co-creators of knowledge, ensuring that their voices were central to the themes and insights generated. Additionally, the researchers—particularly JE and GR—contributed to the creation of knowledge by leveraging their expertise in cognitive science, enactivism, and the BPS model, thus becoming part of a grounded theory knowledge creation as reflexive researchers. It ensured transparency and mitigated potential biases while acknowledging the co-construction of knowledge between researchers and participants [45]. Before data collection, group discussions were held to reflect on interview themes, allowing the research team to identify potential sources of bias and enhance reflexivity, a key component in constructivist grounded theory [46]. These discussions were designed to ensure that the process of data collection and analysis would be transparent, collaborative, and consistent with the constructivist perspective.

Throughout the confirmability process, individual perspectives from the research team were documented and revisited during analysis to ensure findings were grounded in the data and to validate emerging themes. This approach helped to balance reflexivity with rigour, ensuring that the subjective nature of qualitative inquiry did not compromise the validity of the findings, but rather enriched the interpretive process [47]. Reflexive memos were maintained by all members of the research team to document how their interpretations evolved and how their perspectives influenced the analytical process.

This ensured that objectivity was not in conflict with the constructivist approach, as the goal was to maintain reflexive awareness rather than eliminate researcher influence, thus allowing for a more authentic and co-constructed understanding of the data.

**Fig. 1:**
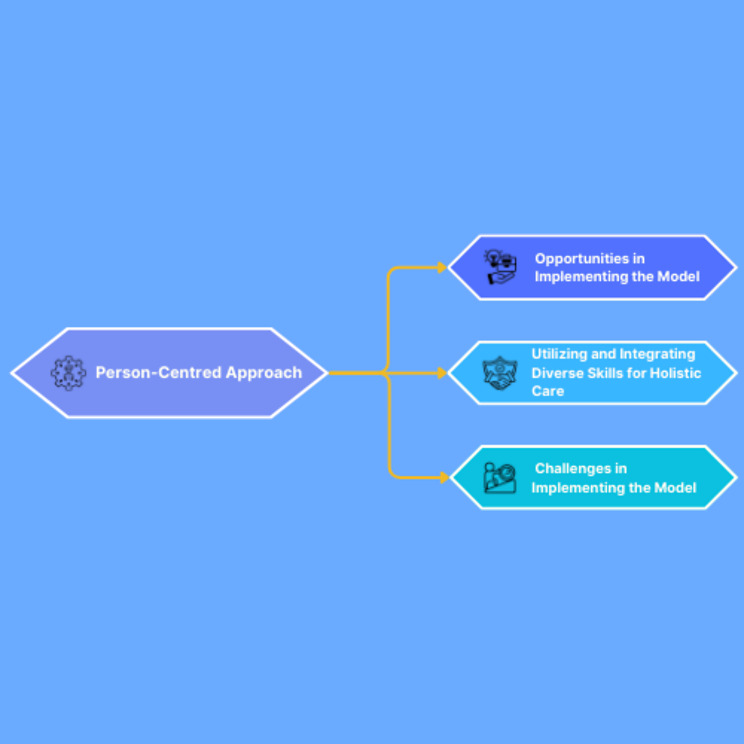
Conceptual map of participants’ beliefs and behaviours using the BPS-E model in CLBP sufferers

Data were sorted into themes using criteria established for Grounded Theory to properly understand the phenomenon [48]. Themes were generated by the principal researcher (MB) and re-explored with other researchers (GR; FC; JE), through multiple rounds of discussion. These discussions allowed for the integration of diverse perspectives, ensuring that the findings were not solely the product of an individual researcher’s interpretation but rather the result of collective analysis. After an exhaustive review, specific codes were assigned to each interview to understand the data obtained [40]. This strategy ensured the confidentiality of respondents by protecting their anonymity. The themes created are illustrated in Fig. 1. The authors employed a rigorous process of member checking, allowing participants to review and verify the themes generated from their interviews, thereby increasing the credibility and trustworthiness of the findings [49]. This process provided an opportunity for participants to affirm the findings, clarify ambiguities, or challenge interpretations. No participant found it necessary to suggest feedback on the findings.

### Trustworthiness

Ensuring trustworthiness in qualitative research is fundamental. Multiple procedures were implemented [35] (see Table 2). The quality criteria used to ensure trustworthiness are based on credibility, transferability, dependability, and confirmability [27].

#### Role of the Funding Source

This research received no external funding.

**Table 2:** Strategies for enhancing and assessing the study’s trustworthiness

Key criteria	Procedures used
**Credibility**	**Supervision and peer evaluation**: the results were continuously reviewed and shared with the other researchers (GR; FC; JE.) and an inspection pathway that used reflexive reminders to allow readers to trace the approach used [30]. Moreover, the comparison with other professionals not part of the project enhanced the credibility of the study. **Member checking**: participants were then able to edit, add or remove any information expressed to increase accuracy. **Data Saturation**: when data gathering and analysis were no longer able to produce novel notions, this cycle of data collecting was deemed to be complete [40, 41]. **The Setting of Interviews**: The remote interview guaranteed the privacy of the participants, the choice of an environment familiar to them, as well as greater flexibility. Furthermore, the interviews were conducted in the absence of other participants to avoid any sort of conditioning [40, 41]. **Preventing Biases**: The authors followed a reflective approach to prevent biases from interfering with the data analysis. Group discussions were held to conduct the reflective process, during which researchers were invited to discuss the subject of the interviews. **Participant Selection**: The heterogeneous and meticulous participant selection allowed readers for easier investigate and interpretation of the results [33]. **Researcher–participant relationship**: The principal researcher (MB) did not have any personal relationship with the interviewees. **Pilot Interview**: it was conducted to focus on the investigation of study themes, and the information gathered in this manner was used to begin the interviews [37].
**Transferability**	**Thorough description**: the e-mail sent to the experienced manual therapists included an exhaustive overview of the research subject to enhance transferability. **Acknowledgement of limits**: the discussion section of this paper includes an in-depth description of the limitations.
**Dependability**	**Control track**: reflection meetings and notes were held to aid analysis and knowledge of how the data were related to the emergent themes.
**Confirmability**	**Researcher’s position**: Because the researcher’s role and experiences affect how data are interpreted and presented in qualitative research, he was regularly supervised by experts with extensive methodological training in this field. **Individual viewpoints**: They were recorded on notes, which were then utilised to check for influences throughout the confirmability process.

## Results

### Participants characteristics and data saturation

Ten out of the twenty contacted professionals participated in the study, representing a variety of clinical work environments and years of clinical experience (Table 3). The group consisted of three physiotherapists, three osteopaths, three individuals with dual qualifications in physiotherapy and osteopathy, and one massage therapist. Their clinical experience ranged from 5 to 10 years for one participant, 10–15 years for two participants, 15–20 years for two participants, and over 20 years for the remaining five participants. The participants’ ages varied from 27 to 66 years, with a mean age of 41.0 years (SD = 6.6).

**Table 3:** Demographic characteristics (*n* = 10)

Variables	Value| *n* (%)
**Age**	41.0 ± 6.6(27–56)
**Gender**	
Male	9 (90)
Female	1 (10)
Non binary/third gender	0 (0)
**Years in Practice**	
< 5 years	0 (0)
5–10 years	1 (10)
10–15 years	2 (20)
15–20 years	2 (20)
> 20 years	5 (50)
**Location of Registration**	
Europe	9 (90)
non-Europe	1 (10)
**Educational Background**	
Osteopath	3 (30)
Physiotherapist	3 (30)
Physiotherapist and Osteopath	3 (30)
Massage therapist	1 (10)
How many professionals have published in peer-reviewed journals on the topics of the BPS model and/or LBP?	5 (50)
How many professionals teach or have taught these topics within an academic context?	8 (80)

Legend: Values are expressed as n(%) and * mean ± standard deviation (range)

The remaining ten manual therapists declined the invitation for personal, time-related, or other unstated reasons. Data saturation was reached after the tenth interview, as no new themes emerged (Table 4). The interviews were conducted over two months, with durations ranging from 45 to 60 min, allowing for a thorough examination of the theme and capturing diverse expert perspectives, while respecting participants’ time constraints.

**Table 4:** Interviews’ saturation process

Interview number	1	2	3	4	5	6	7	8	9	10	Tot
**New themes**	18	11	5	10	12						56
**New themes in rum**						3	2	1	2	0	8
**% change over base**							8,9%		5,3%	3,6%	

### Main theme and subthemes

The analysis revealed one main theme, “The Person-Centred Approach,” with four sub-themes (Fig. 1). These sub-themes, along with illustrative quotes from participants, are described below. Participants’ quotes are identified by numbered square brackets.

### Main theme: the person-centred approach

Subthemes:

1. Opportunities in implementing the model.
2. Utilizing and integrating diverse skills for holistic care.
3. Challenges in implementing the model.

### Main theme: the person-centred approach

The Person-Centred Approach is a holistic framework that emphasizes the person as an active participant in their care. It integrates biological, psychological, and social dimensions to tailor interventions to individual needs. In this study, the Person-Centred Approach serves as the main theme, aligning with the principles of the BPS-E model.

*“…The BPS-E model has fostered a more holistic and person-centred approach to patient care*,* addressing not only the complex interplay of biological*,* psychological*,* and social dimensions but also empowering individuals to take an active role in their care and lives.” [P6]*.

### Subtheme 1: opportunities in implementing the model

Participants recognized various opportunities in implementing the BPS-E model, which combines the holistic aspects of the BPS model with the enactive approach’s focus on the dynamic interplay between individuals and their environments.

The BPS model allows for a comprehensive assessment that goes beyond just the biological aspects of CLBP. It incorporates psychological and social factors, providing a more rounded understanding of the patient’s condition [P1-P10] (see Table 5):

‘*… It allows me to make a holistic assessment of all the different aspects that may influence the patient’s clinical condition.*’ [P7].

Participants noted that integrating the enactive perspective into the BPS model (BPS-E) could lead to improved clinical outcomes. This approach emphasizes the active engagement of patients with their environment, fostering a deeper understanding of their condition and promoting more effective management strategies [P2, P3, P5, P6, P10] (see, Table 5):

‘*…It is necessary to consider the limitations of the BPS model. The enactive approach could be the solution to further improve clinical results.*’ [P1].

The BPS-E model empowers patients by involving them actively in their care process. This engagement helps patients develop a sense of self-efficacy and ownership over their health, which is crucial for long-term management of chronic conditions like CLBP [P1, P3, P5, P6, P7, P10] (see Table 5):

‘*… It is always the patient who holds the keys to his/her destiny and health.*’ [P8].

The BPS-E model highlights the dynamic interaction between individuals and their environment, offering a nuanced understanding of how cognition and physical states are interlinked. This perspective helps therapists tailor interventions that consider the broader context of the patient’s life, leading to more personalized and effective treatments [P1, P3, P5, P6, P10] (see Table 5):

‘*…By considering the patient’s environment and their active engagement with it*,* we can design more effective treatment plans.*’ [P6].

**Table 5:** Quotes. Opportunities in implementing the Model

P1	*“My patients now have more confidence that they can perform normal daily actions that used to scare them. We must not only consider the pain but also the context in which our patients live affects their condition, understood this we will see patients improve.”*
P2	*The patient is not surprised. Often*,* there’s amazement in seeing that you have a different approach from what everyone else has tried*,* and that’s something we*,* who follow this method*,* should value because we live in a world where everyone does the same thing.”*
P3	*“The strengths are clear. I believe the real strength lies in seeing the whole picture of the patient—not just the body*,* not just the joints*,* not just the tissues. […] It has definitely improved my clinical outcomes”*
P4	*“You need to respect the patient’s preferences. If someone strongly believes in the role of manipulation*,* you can probably try it. It plays a role in neurophysiological and psychosocial modulation. If someone is not comfortable talking*,* you must respect that as well.”*
P5	*“With a patient who tends to guard a lot*,* through a hands-on approach*,* I can try to help them understand what it means to relax. Moreover*,* I might teach techniques they can use at home. This creates a window of opportunity for them to feel freer. […] It’s a model used across many professions*,* leading to a common language. […] I believe it relates much better to how an individual interacts with their environment*,* and also how the environment can impact them. It helps me understand their contextual*,* social*,* family*,* and socio-economic situation.”*
P6	*“For example*,* consider an athlete who has suffered a knee ligament injury. Of course*,* the biological factor is crucial*,* but during the healing process*,* other aspects also become significant*,* as training and playing are social activities. Mood can fluctuate due to biological factors*,* such as endorphins. […] Ultimately*,* you access the person through their physical side*,* which can enable you to connect with other dimensions of that individual.”*
P8	*“The strengths lie in viewing the patient not as a body segment but as a whole*,* allowing us to recognize the uniqueness of each patient. This way*,* we can see three cases of lower back pain (LBP) behaving differently. Since I realized its importance*,* my patients are also more satisfied “*
P9	*“Over the years*,* I have refined my way of working. I believe I have always used this frame of reference. I’ve probably begun to not neglect biology but to re-evaluate it in my decision-making process*,* making it a bit less burdensome.”*
P10	*“I’m not saying that one is better than the other*,* because it’s not*,* but the enactive model*,* with the integration of the environment*,* could offer added value.”*

### Subtheme 2: utilizing and integrating diverse skills for holistic care

The adoption of the BPS-E model emphasizes the importance of integrating a broad range of skills in the clinical management of CLBP, highlighting the cohesive application of effective communication, empathy, patient engagement, manual therapy, exercise, and patient education.

Many participants noted that the BPS-E model has become an ingrained, unconscious standard practice in their clinical approach, reflecting a growing understanding of multifaceted nature of health and acknowledging the significance of biological psychological, and social factors in shaping patients’ experience and outcomes [P1, P2, P7] (see Table 6):

‘*…I have never introduced a model in my practice. Patients needed to remove their disbelief*,* and I needed to go beyond the limits of hands-on care. Now I can help many more patients.*’ [P2].

Interpersonal abilities such as verbal and non-verbal communication, empathy, and active listening were frequently emphasized for building trust and rapport with patients. Participants highlighted their use of these skills to establish a therapeutic alliance, understand patients’ experiences and social contexts, and foster self-efficacy [P1-P10]. This involves reassuring patients, particularly those with chronic conditions, about their ability to manage their pain and emphasizing that the primary responsibility for health lies with them [P1, P3, P5, P6, P7, P10] (see Table 6):

‘*…I use my clinical examination to communicate with patients. For example*,* if a patient manages to do a heavy exercise and then has pain in daily activities*,* I ask him: “Why do you think this happens?”*’ [P1].

‘…It is essential to listen to the patients and reassure them with verbal and non-verbal communication.’ [P3].

‘…The non-verbal strategies and the establishment of trust facilitated the development of an effective therapeutic alliance.’ [P6].

Technical and clinical competencies such as manual therapy, exercise therapy, and patient education were identified as essential components of the BPS-E model. Manual therapy was highly valued for its combined psychological and physiological benefits: it alleviated physical pain while fostering empathy and a sense of caring.

*‘…The problem is to forget our main weapon*,* the touch. Not only because of the effectiveness of the physiological mechanisms but also for its psychosocial components.’ [P10]*.

Exercise therapy was highlighted for its role in patient empowerment and pain control, with participants tailoring programs to individual needs to help patients regain confidence in their bodies and foster control over their health [P1-P10] (see Table 6):

*‘…We must recognize that the inability to carry out our daily activities comes at a price. Similarly*,* following the prescribed exercises also involves a cost. However*,* there are occasions when the pain is not as bad as the solution.’ [P4]*.

Education emerged as a cornerstone of the model, aimed at dismantling misconceptions about CLBP and structural issues, and promoting self-efficacy through understanding biopsychosocial factors [P1, P3, P5, P7, P10] (see Table 6):

*‘…Generally*,* patients with CLBP tend to attribute their problem to something purely structural. It is essential to dismantle this belief.’ [P7]*.

By integrating these diverse skills, participants stressed the importance of creating a holistic and patient-centered treatment plan. This ensures that manual therapy, exercise therapy, and education are not delivered in isolation but as part of a comprehensive strategy that includes empathy, active listening, and personalized communication. Participants noted that this approach does not require additional time per session but often reduces the overall frequency of visits, as patients become more engaged and proactive in their care [P1-P10] (see Table 6):

*‘…By integrating manual therapy with education and psychosocial support*,* we can offer a more holistic and effective treatment for CLBP patients.’ [P6]*.

*‘…The model reduces a lot the number of sessions and increases the empowerment of the patient.’ [P8]*.

**Table 6:** Quotes. Utilizing and integrating diverse skills for holistic care

P2	*…’ We have to learn together how to deal with uncertainty, we both don’t know what is going to happen. I use motivational interviewing a lot. Now I’m moving to the fact that pain is reduced when you give less strength.”*
P3	…*’ To me*,* it is important to set up some goals*,* some very little goals*,* go step by step*,* grow confidence with them*,* and break some barriers in their minds. So it is not just about talking. They have to experience that what you have said*,* it’s correct.’*
P4	…*’ I take great care of the relational aspect. I explain certain mechanisms to the patient using models that are understandable to him*,* for example*,* I try to translate and facilitate some of the patient’s messages*,* I try for empathic communication and to realise an approach based and centred on the patient and considering his point of view.’*
P5	*…’ I would try to use open-end questions and affirmations*,* summaries*,* trying to use their own words*,* trying to elicit their fear*,* trying to understand their expectations. […] I try to explain to them that MRI changes do not predict current pain or the future of back pain’ […]’ A lot of them come from word of mouth*,* have been to many clinicians before seeing me*,* so they decided to come and see me because of my approach. […]’ So I do a lot of education because I think it’s quite key if we want people to be autonomous. But I also think that pure education is not very effective with patients. Experience is a really strong way of educating patients*,* sort of learning by doing sort of thing. Regarding manual therapy*,* it all depends on their previous experience. Therefore they might be not interested at all in that. We have to be mindful that patients with persistent pain do not like doing exercises and they feel that it’s very*,* they feel guilty about not liking exercises. So we have to be quite careful about how we approach these strategies.’ ‘*
P6	*…’ You need to create conditions where you’re kind of surprised by the system positively and develop a very robust relationship with the person*,* what is known as Alliance. Of course*,* it’s not gonna happen with everybody. So that’s life*,* that’s the nature of human relationships. People would say*,* communication*,* is just a soft skill. There are key skills. […] So it’s not just words. Sometimes with complex*,* I just test a few hypotheses. Let’s just see how this feels because that helps me also helps us to understand if I actually*,* can help you or not.’ […]’ I try and avoid pure passive care. So*,* manual therapy for me does have an important role for sure. It is a sort of a vehicle to get to try to understand better the person”*
P7	.*’ I try to give him some confidence in being able to handle the problem. I then try to use very simple examples to be able to explain why it hurts. I try to explain to them there is nothing unsolvable.‘[…]’ Generally*,* patients with chronic low back pain tend very much to attribute their problem to something purely structural at the level of the spine. It is therefore essential to dismantle this belief. […]Manual therapy in my clinical practice*,* to this day I use it a lot as a desensitisation tool*,* mainly to try to reduce the patient’s reactivity.’*
P8	*…’ I try to use*,* more neutral words. I avoid those words that might create an image inside of me of things being strangled or bone*,* or so I avoid those scenarios in their minds. I try to correlate what they feel with things that they have some control over in their daily life.’*
P10	*…’ My focus is on the person itself and I try to make them understand how sometimes X-rays do not show exactly what’s going on in the body. […]’ I always explain to them that low back pain is just a name and is not always directly proportionate to the degree of the lesion. So I try always to make them understand that in the higher percentage*,* this pain is not dangerous.’*

### Subtheme 3: challenges in implementing the model

Despite the potential benefits of the BPS-E model, participants identified several challenges in implementing this person-centred approach in their clinical practice. These challenges include practical barriers, professional boundaries, knowledge gaps, and educational shortcomings.

Participants highlighted various socio-cultural and economic barriers that hinder the implementation of the BPS-E model. These barriers often include unsupportive working conditions, such as limited session durations and high patient volumes, which make it difficult to practice a comprehensive and empathetic approach [P1, P2, P3, P7] (see Table 7):

‘*…There are so many socio-cultural and economic barriers. If I work in a place where the sessions last 20 minutes*,* then I can’t do these things*,* and sometimes patients don’t want to waste time talking.*’ [P2].

Therapists often felt that addressing the psychosocial aspects of the BPS-E model could encroach on the role of a psychologist. They expressed concerns about stepping beyond their professional boundaries and expertise, particularly when dealing with complex psychological issues [P6, P7, P9] (see Table 7):

‘*…Except for psychological clinical conditions that require therapeutic support from a specialized professional*,* I don’t believe that one needs to be a psychologist to be an empathetic human being*.’ [P1].

A significant challenge identified was the lack of comprehensive training on the BPS-E model in both academic and clinical settings, as observed by the participants in their interactions with students and less experienced colleagues. Therapists noted that this gap in knowledge often leaves newer practitioners ill-equipped to effectively identify, address, and manage the psychosocial aspects of patients with chronic conditions. This systemic issue can lead to oversimplification and potential misapplication of the model [P1–P10] (see Table 7):

‘*…We have no training on how to use these tools. There is the risk of just placing the patient in one of the bubbles and excluding the others depending on the patient.*’ [P3].

Participants reported significant gaps in the teaching of how to effectively apply the BPS-E model in clinical practice. This deficiency in education can result in confusion among students and therapists, who may struggle to integrate the model into their practice. Additionally, some therapists may attempt to act as psychologists without achieving positive outcomes, further complicating the effective implementation of the model [P1-P10] (see Table 7):

‘*…The challenge is to be able to design a treatment that responds to EBM and the patient’s expectations.*’ [P5].

Therapists often lack confidence in their ability to address psychosocial elements, partly due to inadequate training and partly due to the perceived complexity of these aspects. This lack of confidence can lead to a reductionist approach, where therapists focus predominantly on the biological components of CLBP, thereby missing the holistic picture [P1, P2, P3, P7] (see Table 7):

‘*…There are so many socio-cultural and economic barriers. If I work in a place where the sessions last 20 minutes*,* then I can’t do these things*,* and sometimes patients don’t want to waste time talking.*’ [P2].

Managing patient expectations was another challenge highlighted by participants. Patients often have preconceived notions about their condition and the treatment they should receive. Educating patients and aligning their expectations with the BPS-E model’s holistic approach requires time and effort, which can be challenging in a busy clinical setting [P1, P3, P5, P7, P10] (see Table 7):

‘*…Generally*,* patients with CLBP tend to attribute their problem to something purely structural. It is essential to dismantle this belief.*’ [P7].

**Table 7:** Quotes. Challenges in implementing the model

P1	*…’ In Portugal, the system is pure biomedical. So, if your opinion is a little bit different or you apply the BPS model, then it is very difficult to change.’*
P2	…*’ The negative is that it is sometimes oversimplified. We know that many things are associated with pain. The problem is that these associations don’t necessarily help us in the treatment. Changing factors such as weight or stress don’t always reduce pain.’*
P3	…*’ If we talk about osteopathy specifically. We are told to remain classical and we are afraid of change, or we have some difficulties to change.’*
*P4*	…*’ We’re trained on manual therapy*,* the exercise and we’re starting while on the psychological aspect we still sometimes act as psychologists*,* but if you don’t have a solid background built up over years you will fail.’*
*P6*	*…’ I’m not a psychologist or I can’t do any intervention from a social perspective. If someone has a job they hate*,* tell them to quit the job it’s easier said than done. Many people say just*,* it’s not within my scope of practice.’*
*P7*	…*’ Changing something ingrained requires a greater commitment on the part of the practitioner*,* a greater loss of time*,* and a greater conversation with the patient that perhaps even in many working conditions you are not allowed to have. […] The student struggles to find the practicalities of the model itself*,* which is perhaps presented in a very abstract way. There is no explanatory part that allows us to understand what psychosocial factors are and how they should be managed ‘*
*P9*	*…’ They come from a session of what they think would be just mostly manual therapy and suddenly they’re sitting on psychiatrists*,* and they don’t want to feel like that sometimes.’*
*P10*	…*’ Without a deep understanding of all areas*,* we can fail and probably areas can also be developed a little more. I think it’s important that it’s taught at some point in education.’*

## Discussion

### Main findings and comparison with evidence

This study aimed to explore the insights of ten manual therapists regarding the implementation of the biopsychosocial-enactive (BPS-E) model in the management of chronic low back pain (CLBP). The participants included three physiotherapists, three osteopaths, three professionals with dual qualifications in physiotherapy and osteopathy, and one massage therapist. This diverse composition provided a comprehensive perspective on the integration of the BPS-E model into manual therapy clinical practice. The data collection process aligns with previous qualitative research exploring the integration of the BPS model in pain management contexts while expanding it by introducing the enactive perspective [18, 50–52]. The use of snowball sampling facilitated the recruitment of a diverse group of manual therapists with specific expertise in applying the BPS-E model to CLBP management [12, 52]. The findings highlight the necessity of moving beyond the reductionist biomedical model when treating patients with CLBP, a notion supported by prior studies [12, 13, 18, 23–25].

This study initiates an important dialogue regarding the application of the BPS-E model in CLBP care. The results highlight the limitations of the traditional BPS model, as voiced by participants who expressed skepticism about its capacity to fully capture the interconnected nature of biological, psychological, and social factors. Some participants felt that while the BPS model offers a framework to understand pain, it remains limited due to its fragmented application, which aligns with existing critiques [12, 15–18, 23–25]. The reductionist approach and confusion about how to integrate the different domains of pain management have led to calls for more comprehensive models, such as the BPS-E model [12, 13].

The BPS-E model provides a more dynamic and holistic framework by addressing the continuous interactions between individuals and their environments, helping overcome the reductionism inherent in the traditional BPS model [19]. Participants in this study suggested that an enactive approach offers a more flexible understanding of the interplay between body, mind, and environment, avoiding the premature attribution of CLBP to a single cause and instead promoting a comprehensive exploration of pain progression through a biopsychosocial-enactive lens [12].

This broader and more integrated perspective also calls for a reevaluation of how professional skills are conceptualized and applied in clinical practice. Our findings contribute to the ongoing critique of the “hard” and “soft” skills dichotomy, as highlighted in social science literature. Authors such as Continisio et al. (2021) and Dunivin et al. (2020) have highlighted how this binary reflects outdated assumptions, with “hard” skills often associated with masculinity and technical expertise, while “soft” skills are linked to femininity and emotional labour [54–56]. Such categorizations risk undervaluing the critical contributions of “soft” skills in professional contexts, particularly in fields like healthcare, where relational and emotional competencies are essential [53]. However, the participants in this study frequently described skills in ways that challenged this binary, highlighting their interdependence in clinical practice. For example, technical proficiency was often contextualised within relational competencies, such as empathy, active listening, and effective communication, which were often framed as integral to technical tasks, rather than as auxiliary or secondary traits, to deliver effective, patient-centered care. This integration underscores the necessity of moving beyond the “hard/soft” dichotomy to adopt a more holistic and context-dependent understanding of professional skills.

The data also reveal growing consensus among participants that psychological and social factors play a significant role in determining the outcomes and progression of chronic pain [57]. Therapists recognised the importance of identifying and addressing these factors to improve CLBP management, which is consistent with previous findings that underscore the need for holistic treatment approaches [58]. Participants noted that applying the BPS-E model can enhance self-efficacy and build a strong therapeutic alliance by focusing on the whole person, not just their biological systems [57]. This finding supports the growing view that the BPS-E model enables more patient-centred care while promoting recovery and reducing “yellow flags”—psychosocial factors that may increase the risk of prolonged pain [18].

Contrary to concerns raised in other studies [15], participants in this research noted that using the BPS-E model does not necessarily increase the time required for patient care. Instead, they suggested that by incorporating both soft and hard skills, the BPS-E model can improve time efficiency and treatment effectiveness without compromising the quality of care [12, 59]. Each session, lasting 45–60 min, enables therapists to deliver comprehensive care while building strong therapeutic alliances through effective communication and hands-on interventions [18]. Participants also noted that the BPS-E model should not be viewed as a passive practice paradigm but rather as an active one, where hard skills play an important role in communicating with and reassuring patients. A well-reasoned manual intervention, coupled with appropriate verbal and non-verbal communication may putatively alter the patient’s understanding of their condition [15] and strengthen the therapeutic alliance, which is the initial step in improving the quality of care [60, 61].

However, participants, in line with other studies [15–17, 20, 52], also acknowledged that the successful application of the BPS-E model depends on adequate training and confidence, which many therapists felt they lacked. This highlights the importance of embedding the BPS-E model and its principles in healthcare education to better prepare future practitioners for holistic assessments and treatments [19].

### Implications for clinical practice and research

In summary, this study provides valuable insights into the application of the BPS-E model in managing CLBP. It underscores the model’s potential to overcome the limitations of the traditional BPS framework by addressing the complex interplay of biopsychosocial factors through an enactive approach. Moreover, the importance of integrating diverse skills in fostering self-efficacy, building therapeutic alliances, and improving outcomes in CLBP management is highlighted.

However, the gender composition of our participant pool, with nine men and one woman, represents a notable limitation. This imbalance may have influenced the way skills were described, with narratives potentially reflecting a predominantly male perspective. The underrepresentation of women might have constrained the diversity of viewpoints, particularly concerning experiences often linked to relational competencies, which are traditionally undervalued but essential in BPS-E practices.

Future research should address this limitation by incorporating a more balanced gender representation and exploring how individuals of diverse backgrounds conceptualize and integrate these skills in practice. This would further illuminate how traditional narratives about technical and relational skills can be dismantled, fostering a more inclusive understanding of professional competencies. Additionally, future studies could also address additional limitations of this research, including the small sample size and the reliance on self-reported data, which may introduce bias. Triangulating these findings with observational data or objective performance metrics could further enhance the understanding of how diverse skills contribute to effective clinical practice. By critically engaging with these issues, researchers and practitioners alike can contribute to more inclusive and comprehensive frameworks for understanding and developing professional competencies in healthcare.

Future research should continue to explore the practical implementation of the BPS-E model and its impact on clinical outcomes. Furthermore, a more thorough grasp of the model’s real-world application and effects on patients and clinicians would also be possible by including perspectives from patients who suffer from chronic low back pain as well as specialists in the BPS-E model. In addition to enhancing the knowledge acquired from physicians, investigating patient experiences would aid in validating the model’s efficacy and suitability in practical contexts.

### Strengths and limitations

This study is, to our knowledge, the first to qualitatively explore the role of the BPS-E model in the management of CLBP. It provides a preliminary exploration that initiates a valuable debate on the meaning and application of the BPS-E model to improve care for individuals with CLBP. The study’s limitations include a predominantly male participant group (9:1), which may skew interpretations of the results. Furthermore, our findings underscore the inadequacy of the “hard/soft” distinction in capturing the complexity of skills required in healthcare. By framing these competencies as integrated and context-dependent, our study offers a more nuanced understanding of professional practices. This approach aligns with contemporary critiques, which advocate for a holistic perspective that values all dimensions of skilled behaviour, irrespective of their historical gendered connotations. The small sample size further limits a comprehensive analysis of variations in the BPS-E model. Additionally, the European-centric participant distribution (9/10) restricts insights into global model applications. These constraints underline the need for cautious result interpretation, considering participant gender bias, sample size, and geographical concentration.

Furtheremore, this study primarily centered on exploring the BPS-E model and its theoretical implications rather than practitioners’ lived experiences of its implementation. As such, we recognize the critique that this approach may have led to a model-centric interpretation of the findings, which contrasts with the experiential focus typically associated with grounded theory. While we aimed to highlight how the BPS-E model is conceptualized and applied, future research should consider using complementary theoretical frameworks, such as phenomenology or CFIR, to investigate the nuanced decision-making processes and experiential differences among practitioners, particularly in the context of treating CLBP. Finally, while the BPS-E model is proposed as a framework to enhance the traditional BPS model, we recognize that empirical evidence directly comparing the two models in terms of implementation and outcomes is currently limited. Future research should focus on rigorous comparative studies to validate the efficacy of the BPS-E model.

## Conclusion

This study provides valuable insights into the experiences of manual therapists implementing the BPS-E model in managing CLBP. The findings underscore the potential of the BPS-E model to address the limitations of the traditional BPS model by offering a more holistic and dynamic approach to patient care. However, the study also highlights significant challenges in implementing this model, particularly related to professional training, knowledge gaps, and practical barriers.

To overcome these challenges, the study suggests that ongoing professional development and enhanced pre-registration education are essential. These initiatives could better prepare healthcare professionals to adopt a person-centred, rather than a pain-centred, approach to treatment. By focusing on the whole individual and their interactions with their environment, the BPS-E model promotes a more comprehensive and empathetic approach to managing CLBP.

Furthermore, this study initiates a valuable discussion about the significance and practical application of the BPS-E model in clinical settings. The preliminary data highlight the need for further research to explore the model’s effectiveness and to develop strategies for its broader implementation. By addressing these areas, the healthcare field can move towards a more integrated and effective approach to chronic pain management, ultimately improving patient outcomes and quality of life.

## Data Availability

The data that support the findings of this study are available from the corresponding author upon reasonable request.

## Abbreviations

BPS: Biopsychosocial
BPS-E: Biopsychosocial-Enactive
CLBP: Chronic Low Back Pain
EBM: Evidence Based Medicine
GPs: General Practitioners
COREQ: Consolidated Criteria for Reporting Qualitative Research
